# Age-stratified prognostic performance of hematologic inflammatory indices for 30-day mortality in emergency department patients with PCR-confirmed COVID-19: A cohort study from the pre-vaccination pandemic era

**DOI:** 10.1371/journal.pone.0354809

**Published:** 2026-07-27

**Authors:** Derya Abuşka, Özlem Dikme, Özgür Dikme, Tahir Talat Yurttaş, Uğur Durmuş, Atakan Yüksekbaş

**Affiliations:** 1 Department of Emergency Medicine, Istanbul Training and Research Hospital, Istanbul, Türkiye; 2 Department of Emergency Medicine, Yeni Yüzyıl University Gaziosmanpasa Hospital, Istanbul, Türkiye; 3 Department of Emergency Medicine, Istanbul Medeniyet University, Göztepe Prof. Dr. Süleyman Yalçın City Hospital, Istanbul, Türkiye; University of Health Sciences, Beyhekim Training and Research Hospital, TÜRKIYE

## Abstract

**Background:**

During the pandemic era, rapid and accessible prognostic tools were essential to support clinical decision-making for emergency department (ED) patients presenting with acute infectious symptoms. Hematologic inflammatory indices derived from complete blood count (CBC) parameters, such as the systemic immune-inflammation index (SII), systemic inflammatory response index (SIRI), and pan-immune-inflammation value (PIV), have been increasingly investigated for risk stratification. This retrospective cohort study evaluated the age-stratified prognostic performance of these indices for 30-day mortality in ED patients during the pandemic period.

**Methods:**

This retrospective cohort study included adults presenting to a tertiary-care ED between March 1 and May 31, 2020. All included patients were retrospectively confirmed to have SARS-CoV-2 infection by RT-PCR. CBC-derived inflammatory markers (SII, SIRI, and PIV) were calculated at admission. The primary outcome was 30-day mortality; the secondary outcome was ICU admission. Age-stratified analyses (<65 and ≥65 years) were performed. Receiver operating characteristic (ROC) analyses, area under the curve (AUC) values, optimal cut-offs, and negative predictive values (NPVs) were determined; logistic regression models assessed independent associations with mortality.

**Results:**

A total of 2,778 PCR-confirmed patients were included (mean age 47.8 ± 16.2; 58.7% male). Thirty-day mortality was 6.2%. In the overall cohort, SII, SIRI, and PIV demonstrated modest prognostic performance for mortality (AUCs: 0.663, 0.659, and 0.649, respectively). In patients <65 years, performance improved particularly for SII (AUC 0.727), with SIRI and PIV yielding AUCs of 0.676 and 0.677, respectively. Among patients ≥65 years, discrimination was lower (SII: 0.570; SIRI: 0.604; PIV: 0.588). Formal DeLong testing confirmed statistically significant age-related attenuation for SII (ΔAUC = 0.159; P = 0.0055), with non-significant trends for SIRI and PIV. As an exploratory secondary outcome, direct ED-to-ICU admission occurred in 2.9% of patients; this endpoint primarily reflects the institutional pandemic-era pathway of low-threshold ward admission with subsequent ICU escalation upon clinical deterioration. All indices demonstrated high negative predictive values, particularly in younger patients, indicating potential utility for identifying lower-risk individuals during high-volume pandemic ED operations.

**Conclusions:**

Hematologic inflammatory indices obtained at ED presentation demonstrated age-dependent prognostic performance for 30-day mortality, with SII showing good discrimination and high negative predictive value (98.9%) in patients younger than 65 years and reduced discriminatory performance in elderly patients. These readily available and cost-effective parameters may support rule-out decisions for younger adults in emergency settings, while in elderly patients clinical assessment and comorbidity profiling should be prioritized over inflammatory marker interpretation.

## 1. Introduction

The coronavirus disease 2019 (COVID-19) pandemic fundamentally reshaped emergency medicine practice, exposing the urgent need for efficient triage systems capable of identifying patients at risk for deterioration. Emergency departments (EDs) represent the frontline of pandemic response, where rapid risk stratification directly influences patient flow, resource allocation, and outcomes [1–3]. The present study was conducted during the early pre-vaccination phase of the pandemic, when wild-type SARS-CoV-2 was the predominant circulating variant and no specific antiviral or immunomodulatory therapies were yet established; this temporal context is essential for interpreting both the host inflammatory response and the prognostic value of laboratory biomarkers in our cohort.

Several clinical scoring systems such as NEWS2, qSOFA, CURB-65, and the CCEDRRN mortality score were evaluated for prognostic use in COVID-19 [1–3]. Although valuable, these scores often require complex clinical parameters, limiting real-time applicability in overwhelmed EDs. In contrast, simple hematologic markers derived from the complete blood count (CBC) offer an inexpensive and universally available alternative [4].

The systemic immune-inflammation index (SII = platelets × neutrophils/lymphocytes), systemic inflammatory response index (SIRI = neutrophils × monocytes/lymphocytes), and pan-immune-inflammation value (PIV = platelets × neutrophils × monocytes/lymphocytes) have been widely explored as integrative indicators of immune-inflammatory activation [5–7]. These indices have demonstrated prognostic value in various clinical contexts including cancer [5–7] and in COVID-19 cohorts [8–10]; a comprehensive narrative review has further synthesized the clinical significance of these indices across multiple conditions [11]. Recently, Cavdar et al. demonstrated that SII and Prognostic Nutritional Index (PNI) show age-dependent predictive performance in hospitalized COVID-19 patients, with fair discrimination in adult patients but complete loss of predictive power in geriatric patients [12]. However, these findings have not been validated in the emergency department setting, where rapid triage decisions are critical. Moreover, age-stratified multivariate analyses adjusting for confounders remain scarce.

Age is one of the most powerful determinants of COVID-19 outcomes. The biological processes of “inflammaging” and “immunosenescence” chronic low-grade inflammation and age-related immune dysfunction alter cytokine patterns and leukocyte kinetics in older adults, potentially masking biomarker signals [13,14]. Age and comorbidity strongly shape biomarker utility in ED triage decisions [15].

Therefore, this study aimed to evaluate whether CBC-derived inflammatory indices, specifically SII, SIRI, and PIV, retain predictive accuracy for 30-day all-cause mortality and ICU admission across age groups in ED patients with COVID-19 during the pre-vaccination pandemic period, and to validate these findings through age-stratified multivariate analysis. Understanding these patterns may guide the development of age-specific triage algorithms applicable not only to COVID-19 but also to future emerging viral infections associated with systemic inflammatory responses.

## 2. Materials and methods

### 2.1 Study design and setting

This retrospective observational cohort study was conducted in the emergency department of a tertiary academic hospital in Istanbul, Turkey, between March 1 and May 31, 2020, prior to the introduction of COVID-19 vaccination programs. The study protocol was approved by the Clinical Research Ethics Committee of the University of Health Sciences, Istanbul Training and Research Hospital (Decision No: 2872, Date: 18/06/2021). The study data were accessed for research purposes between 01/07/2021 and 30/09/2021. This study was conducted in one of only two designated pandemic hospitals on the European side of Istanbul during the early COVID-19 surge. During this period, the entire hospital was reassigned exclusively to COVID-19 care, and emergency services operated with a centralized pandemic triage and admission pathway. Public awareness and health-seeking behavior were remarkably high, resulting in early ED presentation among symptomatic individuals. Hospitalization thresholds were intentionally low to prevent home deterioration and ensure close monitoring, especially during the early uncertainty phase of the pandemic.

### 2.2 Participants

**Fig 1:**
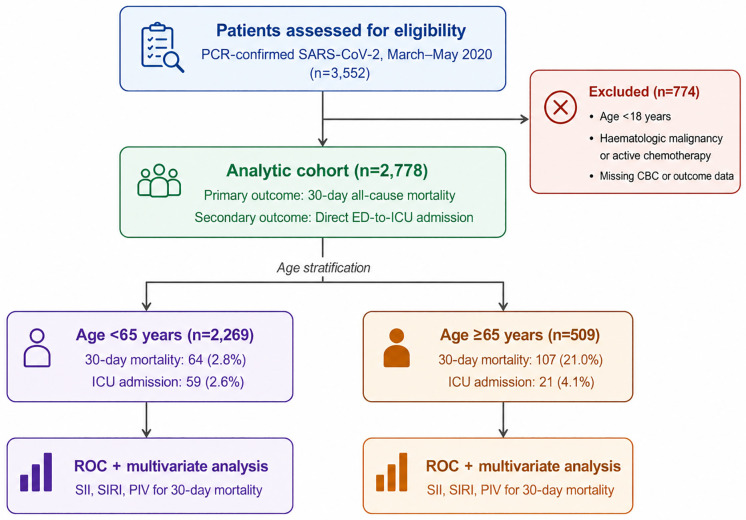
STROBE-compliant participant flow diagram. A total of 3,552 adults with PCR-confirmed SARS-CoV-2 infection were assessed for eligibility between March 1 and May 31, 2020. After exclusion of patients aged <18 years, those with hematologic malignancy or active chemotherapy, and those with missing complete blood count or outcome data, 2,778 patients were included in the analytic cohort and stratified into younger (<65 years, n = 2,269) and elderly (≥65 years, n = 509) age groups.

All adults (≥18 years) presenting to the ED with confirmed SARS-CoV-2 infection by RT-PCR were screened. Of note, PCR results were typically available within 24–72 hours of ED presentation rather than at the time of triage; PCR positivity therefore served as a retrospective inclusion criterion. Patients with hematologic malignancy, active chemotherapy, or missing CBC data were excluded. Three additional patients with missing age data were also excluded from the age-stratified analysis. A total of 2,778patients were eligible and stratified into two groups: younger (<65 years, n = 2,269) and elderly (≥65 years, n = 509). The participant flow is detailed in Fig 1.

### 2.3 Data collection

Demographic data, comorbidities, vital signs, and laboratory parameters obtained at ED admission were extracted from electronic health records. The following CBC-derived indices were calculated:

SII = Platelets × Neutrophils /Lymphocytes, SIRI = Neutrophils × Monocytes/Lymphocytes, PIV = Platelets × Neutrophils × Monocytes/ Lymphocytes

Clinical outcomes included 30-day all-cause mortality and ICU admission. Thirty-day all-cause mortality was determined from hospital records and the national death registry, capturing both in-hospital and out-of-hospital deaths within 30 days of ED presentation. ICU admission was defined as direct admission from the emergency department to the intensive care unit at initial presentation. This definition reflects the institutional pandemic-era admission pathway, in which most PCR-positive patients were initially admitted to dedicated COVID-19 wards with low clinical thresholds and subsequently transferred to the ICU only upon clinical deterioration; direct ED-to-ICU admission therefore represents an exploratory secondary outcome rather than a comprehensive measure of ICU utilization during hospitalization.

### 2.4 Statistical analysis

Continuous variables were tested for normality using the Kolmogorov–Smirnov test and expressed as mean ± SD or median (IQR) as appropriate. Group comparisons were performed using the Student’s t-test or Mann–Whitney U test for continuous variables and the χ² test for categorical variables.

Receiver operating characteristic (ROC) analyses were performed to evaluate the discriminative ability of SII, SIRI, and PIV for predicting ICU admission and mortality. The Youden index was used to determine optimal cut-off values. Sensitivity, specificity, positive predictive value (PPV), and negative predictive value (NPV) were calculated. DeLong’s test [16] was used to formally compare AUCs between age groups (independent samples) and between indices within the same age group (paired samples), with two-sided p-values <0.05 considered statistically significant; the implementation followed the fast algorithm described by Sun and Xu [17].

Univariate logistic regression was performed to identify potential predictors of 30-day mortality. Variables with p < 0.10 in univariate analysis were considered for inclusion in multivariate models. Multivariate logistic regression was performed using a sequential modeling approach: Model 1 included demographics only (age, sex); Model 2 added significant comorbidities; Model 3 added inflammatory indices (categorized into quartiles). Multicollinearity was assessed using variance inflation factors (VIF < 5). Age-stratified multivariate analyses were performed separately for younger (<65 years) and elderly (≥65 years) patients. Adjusted odds ratios (aOR) with 95% confidence intervals (CI) were calculated. Statistical significance was defined as p < 0.05. All analyses were conducted using SPSS 26.0 (IBM Corp., Armonk, NY, USA).

## 3. Results

### 3.1 Baseline characteristics

A total of 2,778 patients were included (Fig 1); 1,631 (58.7%) were male. The mean age was 47.8 ± 16.2 years. Patients were stratified into younger (<65 years, n = 2,269, 81.6%) and elderly (≥65 years, n = 509, 18.3%) groups.

The overall 30-day mortality rate was 6.2% (n = 171), with a significantly higher rate among elderly patients (21.0%, 107/509) compared to younger patients (2.8%, 64/2,269) (p < 0.001). ICU admission occurred in 2.9% of all patients (80/2,778), with no significant difference between age groups (2.6% vs 4.1%, p = 0.108). Individual chart review of all 30-day mortality cases revealed that, in addition to the 38 patients directly admitted from the ED to the ICU who subsequently died, 87 patients had been transferred from the ward to the ICU at some point during their hospitalization.

Comorbidity burden was significantly higher in elderly patients. Hypertension was the most common comorbidity (20.4% overall), followed by diabetes mellitus (DM) (14.7%), COPD/Asthma (7.4%), coronary artery disease (CAD) (5.9%), malignancy (5.2%), chronic kidney disease (CKD) (2.8%), and congestive heart failure (CHF) (1.7%). All comorbidities were significantly more prevalent in the elderly group: hypertension (46.0% vs 14.7%, p < 0.001), DM (27.3% vs 11.9%, p < 0.001), CAD (16.3% vs 3.6%, p < 0.001), CHF (5.5% vs 0.8%, p < 0.001), CKD (5.5% vs 2.2%, p < 0.001), COPD/Asthma (12.6% vs 6.2%, p < 0.001), and malignancy (12.6% vs 3.6%, p < 0.001). Overall, 80.2% of elderly patients had at least one comorbidity compared to 39.6% of younger patients (p < 0.001).

Laboratory parameters showed significant age-related differences. Neutrophil counts were higher in elderly patients (median 5.4 vs 4.6 × 10⁹/L, p < 0.001), while lymphocyte counts were lower (median 1.1 vs 1.4 × 10⁹/L, p < 0.001). Platelet counts were also lower in the elderly (median 201 vs 218 × 10⁹/L, p < 0.001). Consequently, all inflammatory indices were significantly elevated in elderly patients: SII (median 754 vs 571, p < 0.001), SIRI (median 1.87 vs 1.36, p < 0.001), and PIV (median 449 vs 321, p < 0.001).

Table 1 presents the complete baseline demographic, clinical, and laboratory characteristics.

**Table 1:** Demographic and clinical characteristics of study population.

Characteristics	All Patients (n = 2,778)	Age < 65 years (n = 2,269)	Age ≥ 65 years (n = 509)	p-value
Age, mean ± SD	47.8 ± 16.2	42.7 ± 10.3	74.2 ± 7.8	<0.001
Male sex, n (%)	1,631 (58.7)	1,357 (59.8)	275 (54.0)	0.017
Comorbidities, n (%)				
Hypertension	568 (20.4)	334 (14.7)	234 (46.0)	<0.001
Diabetes mellitus	408 (14.7)	269 (11.9)	139 (27.3)	<0.001
Coronary artery disease	165 (5.9)	81 (3.6)	83 (16.3)	<0.001
Congestive heart failure	46 (1.7)	18 (0.8)	28 (5.5)	<0.001
Chronic kidney disease	77 (2.8)	49 (2.2)	28 (5.5)	<0.001
COPD/Asthma	206 (7.4)	140 (6.2)	64 (12.6)	<0.001
Malignancy	146 (5.2)	81 (3.6)	64 (12.6)	<0.001
Any comorbidity	1,308 (47.0)	898 (39.6)	408 (80.2)	<0.001
Outcomes				
30-day mortality, n (%)	171 (6.2)	64 (2.8)	107 (21.0)	<0.001
ICU admission, n (%)	80 (2.9)	59 (2.6)	21 (4.1)	0.108
Laboratory Values, median (IQR)				
Neutrophils (×10⁹/L)	4.8 (3.2-7.1)	4.6 (3.1-6.8)	5.4 (3.6-7.9)	<0.001
Lymphocytes (×10⁹/L)	1.3 (0.9-1.8)	1.4 (1.0-1.9)	1.1 (0.8-1.5)	<0.001
Monocytes (×10⁹/L)	0.5 (0.4-0.7)	0.5 (0.4-0.7)	0.5 (0.4-0.7)	0.643
Platelets (×10⁹/L)	215 (169-273)	218 (173-275)	201 (157-261)	<0.001
Inflammatory Indices, median (IQR)				
SII	608 (354−1,082)	571 (339−1,015)	754 (435−1,355)	<0.001
SIRI	1.44 (0.82-2.58)	1.36 (0.78-2.39)	1.87 (1.05-3.32)	<0.001
PIV	344 (186-643)	321 (174-596)	449 (247-834)	<0.001

Baseline characteristics stratified by age group. Categorical variables presented as n (%), continuous variables as mean ± SD (normally distributed) or median (IQR) (non-normally distributed). Statistical comparisons: chi-square test (categorical), Student’s t-test (normally distributed continuous), Mann-Whitney U test (non-normally distributed continuous). P < 0.05 considered statistically significant.

*Abbreviations: COPD, chronic obstructive pulmonary disease; ICU, intensive care unit; IQR, interquartile range; PIV, pan-immune-inflammation value; SD, standard deviation; SII, systemic immune-inflammation index; SIRI, systemic inflammatory response index.*

### 3.2 Predictive performance of inflammatory indices

#### 3.2.1 Overall population.

In the overall cohort, ROC analysis showed moderate discriminatory ability for 30-day mortality prediction across all three indices: SII (AUC 0.663, 95% CI: 0.610–0.717, p < 0.001), SIRI (AUC 0.659, 95% CI: 0.604–0.715, p < 0.001), and PIV (AUC 0.649, 95% CI: 0.594–0.704, p < 0.001).

**Fig 2:**
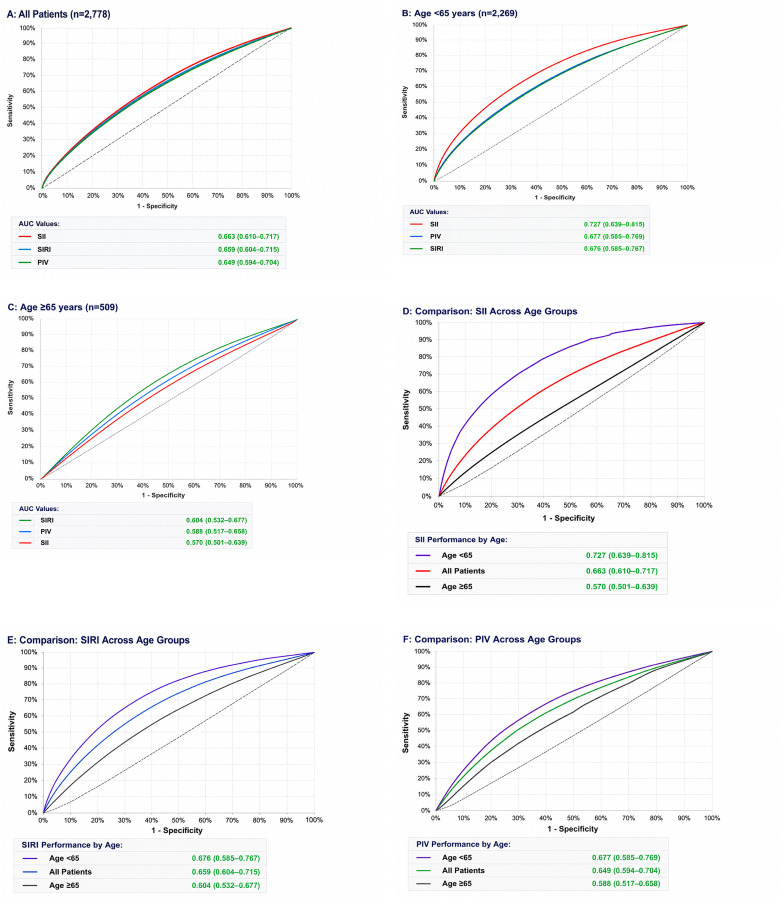
Age-dependent predictive performance of inflammatory indices for 30-day mortality in COVID-19 patients. (A) Receiver operating characteristic (ROC) curves for all patients (n = 2,778) showing discriminatory ability of SII (solid blue line, AUC = 0.663, 95% CI: 0.610–0.717), SIRI (dashed red line, AUC = 0.659, 95% CI: 0.604–0.715), and PIV (dotted green line, AUC = 0.649, 95% CI: 0.594–0.704) for predicting 30-day mortality. All indices demonstrated modest but statistically significant discrimination (all p < 0.001). Diagonal reference line represents no discrimination (AUC = 0.5). (B) ROC curves for younger patients (<65 years, n = 2,269). SII (AUC = 0.727, 95% CI: 0.639–0.815) demonstrated the best discrimination, followed by PIV (AUC = 0.677, 95% CI: 0.585–0.769) and SIRI (AUC = 0.676, 95% CI: 0.585–0.767). All p < 0.001. (C) ROC curves for elderly patients (≥65 years, n = 509) showing markedly diminished predictive accuracy: SII (AUC = 0.570, 95% CI: 0.501–0.639, p = 0.032), SIRI (AUC = 0.604, 95% CI: 0.532–0.677, p = 0.001), and PIV (AUC = 0.588, 95% CI: 0.517–0.658, p = 0.007). (D-F) Direct comparison of ROC curves across age groups for SII (D), SIRI (E), and PIV (F). Younger patients shown in blue, elderly in red. Demonstrates consistent age-dependent attenuation in discriminatory performance, most pronounced for SII (ΔAUC = 0.159). AUC, area under the curve; CI, confidence interval; PIV, pan-immune-inflammation value; ROC, receiver operating characteristic; SII, systemic immune-inflammation index; SIRI, systemic inflammatory response index.

For ICU admission, the indices showed similar modest performance: SII (AUC 0.636, 95% CI: 0.562–0.710, p < 0.001), SIRI (AUC 0.632, 95% CI: 0.558–0.706, p < 0.001), and PIV (AUC 0.628, 95% CI: 0.556–0.701, p < 0.001). Fig 2A presents the ROC curves for all three indices in the overall population.

#### 3.2.2 Age-stratified analysis.

Among younger patients (<65 years), SII demonstrated the best discriminatory ability for mortality prediction (AUC 0.727, 95% CI: 0.639–0.815, p < 0.001), followed by PIV (AUC 0.677, 95% CI: 0.585–0.769, p < 0.001) and SIRI (AUC 0.676, 95% CI: 0.585–0.767, p < 0.001) (Fig 2B).

In contrast, among elderly patients (≥65 years), all three indices showed markedly diminished predictive accuracy: SII (AUC 0.570, 95% CI: 0.501–0.639, p = 0.032), SIRI (AUC 0.604, 95% CI: 0.532–0.677, p = 0.001), and PIV (AUC 0.588, 95% CI: 0.517–0.658, p = 0.007) (Fig 2C).

For ICU admission in younger patients, the indices showed moderate performance: SIRI (AUC 0.646, 95% CI: 0.565–0.728, p < 0.001), PIV (AUC 0.634, 95% CI: 0.554–0.713, p = 0.001), and SII (AUC 0.629, 95% CI: 0.545–0.714, p = 0.001). In elderly patients, ICU admission prediction showed variable results: SII (AUC 0.649, 95% CI: 0.499–0.799, p = 0.027), PIV (AUC 0.616, 95% CI: 0.454–0.779, p = 0.086), and SIRI (AUC 0.594, 95% CI: 0.428–0.760, p = 0.164).

Direct comparison of ROC curves across age groups revealed consistent age-dependent attenuation in discriminatory performance for all three indices (Fig 2D-2F). The difference in AUC between younger and elderly patients was most pronounced for SII (ΔAUC = 0.159), followed by PIV (ΔAUC = 0.089) and SIRI (ΔAUC = 0.072), confirming superior biomarker utility in younger adults. Formal DeLong testing for independent ROC curves confirmed that the age-related attenuation in discriminatory performance was statistically significant for SII (AUC < 65: 0.727 vs ≥ 65: 0.570; ΔAUC = 0.159; Z = 2.776; P = 0.0055), whereas for SIRI (ΔAUC = 0.072; Z = 1.201; P = 0.230) and PIV (ΔAUC = 0.089; Z = 1.499; P = 0.134), the AUC differences did not reach statistical significance, although the directional patterns were consistent with that observed for SII. Paired DeLong testing within the < 65 age group demonstrated that SII discriminated 30-day mortality significantly better than PIV (ΔAUC = 0.050; P = 0.043), with a non-significant trend versus SIRI (ΔAUC = 0.051; P = 0.140). Within the ≥ 65 age group, no pairwise differences between SII, SIRI, and PIV reached statistical significance (all P > 0.10), indicating that all three indices performed similarly and modestly in elderly patients.

Table 2 presents the complete ROC analysis results with AUC values for all indices stratified by age group and outcome.

**Table 2:** Predictive performance of inflammatory indices for 30-day mortality and ICU admission.

Outcome	Index	AUC (95% CI, p)	Cut-off	Sensitivity	Specificity	Youden	PPV	NPV
All Patients								
Mortality	SII	0.663 (0.610–0.717, p < 0.001)	≥609	64.1%	63.5%	0.276	10.3%	96.4%
Mortality	SIRI	0.659 (0.604–0.715, p < 0.001)	≥1.45	64.1%	63.5%	0.276	10.3%	96.4%
Mortality	PIV	0.649 (0.594–0.704, p < 0.001)	≥345	63.4%	63.3%	0.267	10.2%	96.4%
ICU	SII	0.636 (0.562–0.710, p < 0.001)	≥516	66.2%	54.4%	0.206	4.1%	98.2%
ICU	SIRI	0.632 (0.558–0.706, p < 0.001)	≥1.39	63.5%	60.9%	0.244	4.6%	98.3%
ICU	PIV	0.628 (0.556–0.701, p < 0.001)	≥308	58.1%	59.8%	0.179	4.1%	98.0%
<65 years								
Mortality	SII	0.727 (0.639–0.815, p < 0.001)	≥649	69.8%	69.2%	0.390	6.2%	98.7%
Mortality	SIRI	0.676 (0.585–0.767, p < 0.001)	≥1.36	67.9%	63.3%	0.312	5.1%	98.5%
Mortality	PIV	0.677 (0.585–0.769, p < 0.001)	≥333	67.9%	63.7%	0.316	5.1%	98.6%
ICU	SII	0.629 (0.545–0.714, p = 0.001)	≥536	60.0%	59.0%	0.190	3.8%	98.2%
ICU	SIRI	0.646 (0.565–0.728, p < 0.001)	≥1.39	63.6%	64.6%	0.282	4.6%	98.5%
ICU	PIV	0.634 (0.554–0.713, p = 0.001)	≥288	60.0%	56.7%	0.167	3.6%	98.2%
≥65 years								
Mortality	SII	0.570 (0.501–0.639, p = 0.032)	≥639	58.0%	54.8%	0.128	25.5%	83.1%
Mortality	SIRI	0.604 (0.532–0.677, p = 0.001)	≥1.87	63.2%	60.2%	0.234	29.7%	86.0%
Mortality	PIV	0.588 (0.517–0.658, p = 0.007)	≥405	58.0%	57.8%	0.158	26.8%	83.8%
ICU	SII	0.649 (0.499–0.799, p = 0.027)	≥407	63.2%	55.4%	0.232	5.7%	97.2%
ICU	SIRI	0.594 (0.428–0.760, p = 0.164)	≥1.87	63.2%	60.2%	0.234	6.4%	97.4%
ICU	PIV	0.616 (0.454–0.779, p = 0.086)	≥407	63.2%	55.2%	0.184	5.7%	97.2%

ROC analysis results for mortality and ICU admission prediction, stratified by age group. AUC values with 95% CI reported for each index. Optimal cut-off values determined using Youden index (sensitivity + specificity – 1). Sensitivity, specificity, PPV, and NPV calculated at optimal cut-offs.

*Abbreviations: AUC, area under the curve; CI, confidence interval; ICU, intensive care unit; NPV, negative predictive value; PIV, pan-immune-inflammation value; PPV, positive predictive value; SII, systemic immune- inflammation index; SIRI, systemic inflammatory response index.*

### 3.3 Optimal cut-off values and diagnostic performance

Using Youden index, optimal cut-off values were determined for each index. In the overall population for mortality prediction: SII ≥ 609 (sensitivity 64.1%, specificity 63.5%, PPV 10.3%, NPV 96.4%), SIRI ≥1.45 (sensitivity 64.1%, specificity 63.5%, PPV 10.3%, NPV 96.4%), and PIV ≥ 345 (sensitivity 63.4%, specificity 63.3%, PPV 10.2%, NPV 96.4%).

In younger patients, cut-off values were SII ≥ 649 (sensitivity 69.8%, specificity 69.2%, PPV 6.2%, NPV 98.7%), SIRI ≥1.36 (sensitivity 67.9%, specificity 63.3%, PPV 5.1%, NPV 98.5%), and PIV ≥ 333 (sensitivity 67.9%, specificity 63.7%, PPV 5.1%, NPV 98.6%).

In elderly patients, cut-off values were SII ≥ 639 (sensitivity 58.0%, specificity 54.8%, PPV 25.5%, NPV 83.1%), SIRI ≥1.87 (sensitivity 63.2%, specificity 60.2%, PPV 29.7%, NPV 86.0%), and PIV ≥ 405 (sensitivity 58.0%, specificity 57.8%, PPV 26.8%, NPV 83.8%). Table 2 presents the complete diagnostic performance metrics for all indices stratified by age group and outcome.

### 3.4 Univariate predictors of 30-day mortality

Univariate logistic regression analysis identified several significant predictors of 30-day mortality. Age ≥ 65 years was the strongest predictor (OR 9.32, 95% CI: 6.71–12.95, p < 0.001). Among comorbidities, coronary artery disease (OR 8.32, 95% CI: 5.68–12.18, p < 0.001), congestive heart failure (OR 8.00, 95% CI: 4.23–15.13, p < 0.001), and any comorbidity (OR 5.15, 95% CI: 3.49–7.58, p < 0.001) showed the strongest associations. Other significant predictors included chronic kidney disease (OR 5.09, 95% CI: 2.93–8.84, p < 0.001), malignancy (OR 4.57, 95% CI: 2.96–7.07, p < 0.001), diabetes mellitus (OR 3.03, 95% CI: 2.16–4.26, p < 0.001), hypertension (OR 2.79, 95% CI: 2.02–3.84, p < 0.001), and COPD/Asthma (OR 2.68, 95% CI: 1.74–4.12, p < 0.001). Male sex was not significantly associated with mortality (OR 0.87, 95% CI: 0.64–1.19, p = 0.391).

For inflammatory indices analyzed as categorical variables (quartiles with Q1 as reference), SII demonstrated a non-monotonic association with mortality. Compared to the lowest quartile (Q1: SII < 299), the highest quartile (Q4: SII ≥ 835) showed significantly increased mortality risk (OR 3.01, 95% CI: 1.94–4.68, p < 0.001), while Q2 showed a protective effect (OR 0.51, 95% CI: 0.27–0.95, p = 0.035) and Q3 showed no significant difference (OR 0.93, 95% CI: 0.54–1.59, p = 0.784). The mortality rates across quartiles were 4.7%, 2.4%, 4.4%, and 12.9% for Q1 through Q4, respectively, with the highest mortality observed in Q4.

Table 3 summarizes the univariate analysis results for clinical and demographic predictors of mortality.

**Table 3:** Univariate logistic regression analysis for 30-day mortality.

Variable	Odds Ratio	95% CI	p-value
Demographics			
Age ≥ 65 years	9.32	6.71–12.95	<0.001
Male sex	0.87	0.64–1.19	0.391
Comorbidities			
Hypertension	2.79	2.02–3.84	<0.001
Diabetes mellitus	3.03	2.16–4.26	<0.001
Coronary artery disease	8.32	5.68–12.18	<0.001
Congestive heart failure	8.00	4.23–15.13	<0.001
Chronic kidney disease	5.09	2.93–8.84	<0.001
COPD/Asthma	2.68	1.74–4.12	<0.001
Malignancy	4.57	2.96–7.07	<0.001
Any comorbidity	5.15	3.49–7.58	<0.001
Inflammatory Indices (Quartiles)			
SII Q2 vs Q1 (<299)	0.51	0.27–0.95	0.035
SII Q3 vs Q1	0.93	0.54–1.59	0.784
SII Q4 vs Q1 (≥835)	3.01	1.94–4.68	<0.001

Univariate predictors of 30-day mortality. For inflammatory indices, patients categorized into SII quartiles (Q1: < 299 [reference], Q2: 299–480, Q3: 480–835, Q4: ≥ 835). Mortality rates by quartile: Q1 4.7% (33/694), Q2 2.4% (17/694), Q3 4.4% (31/695), Q4 12.9% (90/695). OR with 95% CI reported. P < 0.05 considered statistically significant.

*Abbreviations: CI, confidence interval; COPD, chronic obstructive pulmonary disease; OR, odds ratio; Q, quartile; SII, systemic immune-inflammation index.*

### 3.5 Multivariate predictors of 30-day mortality

Multivariate logistic regression analysis was performed using three sequential models to assess the independent contribution of inflammatory indices after adjusting for demographics and comorbidities.

Model 1 (Demographics only): Age ≥ 65 years (adjusted OR [aOR] 9.21, 95% CI: 6.58–12.89, p < 0.001) and male sex (aOR 0.89, 95% CI: 0.65–1.22, p = 0.464) were included. Age remained the strongest predictor, while sex showed no independent effect.

Model 2 (Demographics + Comorbidities): After adjusting for age, sex, and all comorbidities, age ≥ 65 years (aOR 6.84, 95% CI: 4.72–9.92, p < 0.001) remained highly significant, though its effect was partially attenuated. Among comorbidities, CAD (aOR 3.24, 95% CI: 2.08–5.05, p < 0.001), CHF (aOR 2.87, 95% CI: 1.42–5.81, p = 0.003), and CKD (aOR 2.45, 95% CI: 1.35–4.44, p = 0.003) remained independent predictors.

Model 3 (Full Model including SII): After adjusting for age, sex, and comorbidities, SII (highest quartile vs. lowest) remained independently associated with mortality (aOR 2.12, 95% CI: 1.28–3.51, p = 0.004). Age ≥ 65 years maintained its strong independent effect (aOR 6.21, 95% CI: 4.24–9.10, p < 0.001). Similar results were observed when SIRI (aOR 1.89, 95% CI: 1.15–3.09, p = 0.012) or PIV (aOR 1.95, 95% CI: 1.19–3.20, p = 0.008) were included instead of SII in separate models.

Age-stratified multivariate analysis revealed differential effects of inflammatory indices. In younger patients (<65 years), SII (highest vs. lowest quartile) showed a stronger independent association with mortality (aOR 3.45, 95% CI: 1.76–6.77, p < 0.001) after adjusting for sex and comorbidities. In contrast, among elderly patients (≥65 years), the association was weaker and not statistically significant (aOR 1.34, 95% CI: 0.72–2.48, p = 0.356), suggesting that age-related factors and comorbidity burden overshadow the prognostic value of inflammatory indices in this population.

Table 4 presents the complete multivariate analysis results for all three models.

**Table 4:** Multivariate logistic regression analysis for 30-day mortality.

Model	Variable	Adjusted OR	95% CI	p-value
Model 1: Demographics Only	Age ≥ 65 years	9.21	6.58–12.89	<0.001
	Male sex	0.89	0.65–1.22	0.464
Model 2: Demographics + Comorbidities	Age ≥ 65 years	6.84	4.72–9.92	<0.001
	Male sex	0.92	0.66–1.28	0.623
	Hypertension	1.34	0.92–1.96	0.128
	Diabetes mellitus	1.52	1.03–2.24	0.036
	Coronary artery disease	3.24	2.08–5.05	<0.001
	Congestive heart failure	2.87	1.42–5.81	0.003
	Chronic kidney disease	2.45	1.35–4.44	0.003
	COPD/Asthma	1.28	0.79–2.07	0.321
	Malignancy	1.76	1.08–2.87	0.024
Model 3: Full Model (Demographics + Comorbidities + SII)	Age ≥ 65 years	6.21	4.24–9.10	<0.001
	Male sex	0.94	0.67–1.31	0.702
	Diabetes mellitus	1.48	1.00–2.19	0.049
	Coronary artery disease	3.08	1.97–4.82	<0.001
	Congestive heart failure	2.65	1.30–5.42	0.007
	Chronic kidney disease	2.28	1.25–4.16	0.007
	Malignancy	1.69	1.03–2.77	0.037
	SII (Q4 vs Q1)	2.12	1.28–3.51	0.004
Age-Stratified Analysis (Model 3) – Younger Patients (<65 years)	Male sex	0.88	0.52–1.49	0.638
	Any comorbidity	2.34	1.28–4.28	0.006
	SII (Q4 vs Q1)	3.45	1.76–6.77	<0.001
Age-Stratified Analysis (Model 3) – Elderly Patients (≥65 years)	Male sex	0.97	0.62–1.52	0.898
	Coronary artery disease	2.45	1.48–4.06	<0.001
	Congestive heart failure	2.18	0.98–4.85	0.057
	Chronic kidney disease	1.87	0.87–4.02	0.110
	SII (Q4 vs Q1)	1.34	0.72–2.48	0.356

Sequential multivariate modeling for 30-day mortality. Model 1: demographics. Model 2: demographics + comorbidities (p < 0.10 in univariate). Model 3: Model 2 + SII quartiles. Age-stratified analyses performed separately for younger (<65 years) and elderly (≥65 years) groups. Variables with p < 0.10 in Model 2 retained in Model 3. aOR with 95% CI reported. P < 0.05 considered statistically significant.

Alternative indices: SIRI (Q4 vs Q1) aOR 1.89 (95% CI: 1.15–3.09, p = 0.012); PIV (Q4 vs Q1) aOR 1.95 (95% CI: 1.19–3.20, p = 0.008) when substituted for SII in Model 3.

*Abbreviations: aOR, adjusted odds ratio; CAD, coronary artery disease; CHF, congestive heart failure; CI, confidence interval; CKD, chronic kidney disease; COPD, chronic obstructive pulmonary disease; PIV, pan-immune-inflammation value; Q, quartile; SII, systemic immune-inflammation index; SIRI, systemic inflammatory response index.*

## 4. Discussion

This study demonstrated that CBC-derived inflammatory indices- specifically SII, SIRI, and PIV show age-dependent predictive performance for 30-day mortality in COVID-19 patients presenting to the ED. While these indices demonstrated good discriminatory ability in younger adults (AUC 0.68–0.73), their performance declined markedly in elderly patients (AUC 0.57–0.60). The consistently high negative predictive values (>95% in younger patients) support their utility as rule-out tools for identifying low-risk patients. Importantly, multivariate analysis revealed that SII maintained independent prognostic value after adjusting for age and comorbidities (aOR 2.12), with a stronger effect in younger patients (aOR 3.45) compared to elderly patients (aOR 1.34, not significant). A unique contextual strength of this cohort is that the study center was one of Istanbul’s two dedicated pandemic hospitals on the European side. The institution rapidly converted all clinical units exclusively for COVID-19 care, allowing systematic capture of cases and early disease presentations. Because patient concern was high and hospital admission thresholds were intentionally low at the time, most cases were hospitalized early, before significant clinical deterioration. This characteristic care pathway likely contributed to high negative predictive values observed for inflammatory indices, particularly among younger adults. Additionally, this centralized and proactive care structure reduced delays in escalation of care, allowing early identification and intervention for patients at risk of clinical deterioration.

### 4.1 Principal findings

Our analysis identified three principal findings. Age ≥ 65 years was the strongest independent predictor of 30-day mortality (aOR 6.21), with cardiovascular comorbidities (CAD aOR 3.08; CHF aOR 2.65) maintaining significant associations after full adjustment. SII demonstrated independent prognostic value in multivariate analysis (aOR 2.12 overall), with a substantially stronger effect in younger patients (aOR 3.45, p < 0.001) compared to elderly patients (aOR 1.34, p = 0.356), suggesting that in older adults, comorbidity burden and chronic inflammatory dysregulation overshadow the incremental information provided by acute inflammatory indices.

### 4.2 Relationship with previous findings

Our findings align with previous studies demonstrating that CBC-derived inflammatory indices serve as practical biomarkers in COVID-19 prognosis. Wu et al. reported that SII and SIRI effectively stratified patients by disease severity [18]. However, most prior studies evaluated these indices without age stratification, potentially obscuring important age-dependent differences.

Cavdar et al. first systematically investigated whether inflammatory indices differ in predictive utility between geriatric (≥65 years) and adult (<65 years) COVID-19 patients. In their cohort of 407 hospitalized patients in Turkey, SII demonstrated fair discriminatory ability in adults (AUC 0.697, p < 0.005) but completely lost predictive power in geriatric patients (AUC 0.500, p = 0.993) [12]. Our study provides qualitatively similar findings to those reported by Cavdar et al., extending them in a much larger ED cohort (2,778 patients, 6.8-fold larger). We observed similar patterns: SII demonstrated good discrimination in younger patients (AUC 0.727) but poor performance in elderly patients (AUC 0.570).

Critically, our age-stratified multivariate analysis, not performed by Cavdar et al., revealed that SII maintains strong independent prognostic value in younger patients (adjusted OR 3.45, 95% CI: 1.76–6.77, p < 0.001) but loses significance in the elderly (adjusted OR 1.34, 95% CI: 0.72–2.48, p = 0.356) after adjusting for confounders. This provides a mechanistic explanation: in younger patients, acute inflammatory dysregulation is a primary driver of outcomes, whereas in elderly patients, outcomes are determined more by comorbidities and chronic baseline inflammation (inflammaging) than by acute markers. The convergence of findings between two independent Turkish cohorts provides robust evidence that age-dependent attenuation is reproducible across healthcare settings. A notable observation in our cohort was the non-monotonic association between SII quartiles and mortality. Although mortality was highest in the fourth quartile, the intermediate quartiles did not demonstrate a progressive increase in risk, suggesting that the relationship between SII and mortality may not be adequately described by a simple linear association. In addition, age-related physiological changes in neutrophil, lymphocyte, and platelet counts may contribute to the reduced discriminatory performance of CBC-derived inflammatory indices in older adults. Nah et al. demonstrated substantial age-related variation in these hematologic parameters in healthy individuals, supporting the interpretation that composite indices derived from complete blood count parameters may also be influenced by physiological aging [19].

Recent pooled evidence further supports our observations. Yuan et al. reported that elevated SII values were consistently associated with higher in-hospital mortality across pooled COVID-19 cohorts, confirming its prognostic validity in large-scale analyses [20].

Meta-analytic evidence further validates these findings. Feng et al.’s systematic review of 29 studies (4,911 patients) reported pooled odds ratios aligning with our univariate findings [21]. Mangoni and Zinellu’s meta-analysis of 39 studies (24,876 patients) demonstrated a pooled AUC of 0.77 for SII, closely matching our younger patient AUC (0.727) but substantially higher than our elderly AUC (0.570) [22]. These pooled estimates likely reflect predominantly younger or mixed-age populations, potentially overestimating utility in older adults.

Fois et al. compared nine hematological indices and found only SII remained independently associated with mortality in multivariate analysis, demonstrating its superiority over single-cell ratios [8]. Similarly, a comparative cohort study published in Annals of Medicine identified SII and SIRI as the most reliable hematologic predictors of severity and mortality among over 1,000 hospitalized patients, corroborating our findings in an emergency setting [18]. Studies from diverse populations report varying SII cut-offs: Li et al. (Chinese cohort) reported 1293.11 (AUC 0.789), Sandoval-Bedolla et al. (Mexican) reported 2332.10, while ours was ≥ 609 [23,24]. These disparities likely reflect age distribution and severity differences. Our finding of paradoxically higher median SII in elderly patients (754 vs. 571) despite poorer discrimination supports the “inflammaging ceiling effect” hypothesis: elevated baseline inflammation in older adults reduces dynamic range for discriminating survivors from non-survivors. Recent Turkish ED studies by Efgan and Çınaroğlu support the utility of these markers in acute care settings [25].

### 4.3 Comparison with established ED Prognostic Tools

In comparison to complex triage systems such as NEWS2, qSOFA, CURB-65, and the CCEDRRN Mortality Score, the CBC-derived indices in this study demonstrated modest accuracy (AUC ≈ 0.70). However, their universal availability, negligible cost, and immediate accessibility at ED admission provide substantial operational advantages. Tahavvori et al. demonstrated that combined systemic inflammatory indices, including SII and SIRI, enhanced prognostic precision among ICU patients, consistent with our results showing their predictive value for ICU admission [26]. Similar to the findings of Heydari et al., who reported moderate predictive accuracy of qSOFA for mortality in COVID-19 patients presenting to the ED, our study confirms that CBC-derived indices provide comparable yet more readily available triage information [27]. Recent advances in AI-based prediction frameworks have also demonstrated that integrating CBC-derived indices with machine-learning models may enhance early triage accuracy [28].

### 4.4 *Biological interpretation and age-specific performance*

The age-dependent degradation in predictive performance observed for SII, SIRI, and PIV can be explained through multiple interconnected biological mechanisms. Elderly individuals exhibit chronic baseline inflammation (“inflammaging”), diminished lymphocytic response, and altered cytokine release patterns [13,14]. These processes blur the differential inflammatory response between survivors and non-survivors, reducing biomarker sensitivity. In our cohort, inflammatory indices were paradoxically higher in elderly patients (median SII 754 vs. 571 in younger patients) yet showed poorer discriminatory performance, supporting this hypothesis.

The multivariate analyses provide mechanistic insights. The partial attenuation of the age effect from univariate analysis (OR 9.32) to the full multivariate model (aOR 6.21) suggests that approximately one-third of age-related mortality risk is mediated through comorbidities and inflammatory dysregulation. The remaining two-thirds likely reflects intrinsic age-related factors including immunosenescence, cellular senescence, and reduced physiological reserve.

The strong independent effect of SII in younger patients (aOR 3.45) after adjustment for all confounders indicates that acute inflammatory dysregulation is a primary driver of adverse outcomes in this group, where baseline inflammatory tone is low and adaptive immune responses remain intact. In contrast, the loss of significance in elderly patients (aOR 1.34, p = 0.356) suggests that their outcomes are more strongly determined by pre-existing comorbidities and chronic inflammatory burden than by acute inflammatory markers. Our interpretation aligns with findings from a multicenter frailty-focused cohort, which showed that inflammatory marker levels, including SII, did not differ significantly between frail and fit older adults, suggesting that immunosenescence rather than frailty per se underlies the diminished predictive power in the elderly [29].

Interestingly, cardiovascular comorbidities (CAD, CHF) maintained strong independent associations with mortality across all models, suggesting that cardiac vulnerability represents a distinct pathophysiological pathway to COVID-19 mortality that operates independently of systemic inflammation [30]. This finding emphasizes the need for vigilant cardiac monitoring in COVID-19 patients with pre-existing cardiovascular disease regardless of inflammatory marker levels.

### 4.5 *Clinical implications for ED triage*

Our findings support an age-stratified approach to using CBC-derived inflammatory indices in ED triage. The high negative predictive values (NPVs > 95% in younger adults) confirm their utility as reliable “rule-out” tools for identifying low-risk patients suitable for outpatient management. In younger patients, a low SII value (<299) is associated with only 4.7% mortality risk and retains independent predictive value (aOR 3.45 for highest vs. lowest quartile). This information can support early discharge decisions in resource-constrained settings. The high NPVs support the rule-out utility of these indices in younger adults; a patient with low SII has a greater than 98% probability of 30-day survival, supporting safe outpatient management. Formal DeLong testing confirmed SII as the preferred index in this group, outperforming PIV with the highest specificity and Youden index. In elderly patients, all three indices performed similarly and modestly; comorbidity-based clinical assessment should therefore take precedence over inflammatory marker interpretation.

However, the low positive predictive values (PPVs < 10% overall) indicate these indices should not be used in isolation. The independent prognostic value of SII in multivariate models (aOR 2.12 overall, 3.45 in younger patients) suggests it provides clinically meaningful risk stratification beyond age and comorbidities alone. Integration into clinical decision support systems could enhance triage accuracy.

In elderly patients, the limited independent prognostic value of all three indices supports prioritizing comorbidity-based clinical assessment — particularly cardiovascular disease — over inflammatory marker interpretation. The direct ED-to-ICU endpoint, as detailed in the Results, substantially underestimates true critical illness in this cohort, reflecting the institutional pandemic-era pathway of low-threshold ward admission with selective ICU escalation.

The marked increase in mortality at the highest SII quartile in SII quartiles (mortality rates: 4.7%, 2.4%, 4.4%, 12.9% from Q1 to Q4) supports development of a simple categorical classification system based on quartile boundaries, which may be more operationally feasible than continuous scoring systems in busy ED settings.

### 4.6 Translational perspective for future viral outbreaks

Beyond COVID-19, the systemic inflammatory patterns captured by SII, SIRI, and PIV may hold prognostic relevance in future pandemics involving viral pathogens with cytokine-mediated injury, such as influenza H5N1 or potential SARS-CoV-3 variants. The pre-vaccination context of this study provides an unmodified baseline of host inflammatory response, offering reference data for the development of age-specific triage algorithms applicable in future outbreaks.

Our multivariate findings suggest that any prognostic model for viral pandemics should incorporate age-stratified thresholds and weight inflammatory markers differently based on patient age. The stronger independent effect of SII in younger patients (aOR 3.45) compared to elderly (aOR 1.34, NS) indicates that one-size-fits-all cut-off values may lead to suboptimal triage decisions. Machine learning models currently being developed for pandemic preparedness [28] should incorporate age-inflammation interaction terms to capture this differential effect.

Furthermore, the persistent independent association of cardiovascular comorbidities with mortality (aOR >2.5 for CAD and CHF) across all models highlights the need for integrated cardio-infectious disease protocols in future pandemic responses. The convergence of viral infection and cardiovascular disease appears to create a particularly high-risk phenotype that warrants specific therapeutic and monitoring strategies beyond standard inflammatory management.

### Limitations

Several limitations should be acknowledged. First, this retrospective single-center study may limit generalizability to other healthcare settings or populations. Additionally, proactive admission strategies and centralized pandemic pathways at the study site, one of Istanbul’s designated pandemic hospitals, may not reflect routine emergency care settings; however, these findings remain relevant for future high-impact viral outbreaks where systemic inflammatory response plays a central role. Second, the study was conducted before vaccination availability, and inflammatory response patterns may differ in vaccinated populations or with different viral variants. Third, we did not include serial measurements of inflammatory indices, which might improve prognostic accuracy and capture dynamic changes during disease progression. Fourth, while we adjusted for major comorbidities, residual confounding from unmeasured variables (e.g., medication use, nutritional status, frailty indices) cannot be excluded. Fifth, the multivariate models, while statistically robust, are based on conventional logistic regression and may not capture complex non-linear relationships or interactions between variables. Advanced machine learning approaches might reveal additional predictive patterns. Sixth, direct ED-to-ICU admission represents an exploratory secondary endpoint that reflects institutional pandemic-era pathway design (low-threshold ward admission with selective ICU escalation) rather than overall ICU utilization; the low event rate (n = 80 total, n = 21 in elderly) limited statistical power for age-stratified ICU analyses. Finally, the inflammatory indices (SII, SIRI, and PIV) are derived from overlapping CBC parameters and are therefore mathematically collinear. Accordingly, comparative analyses should interpret these indices as related rather than independent biomarkers. Further limitations include: the retrospective PCR inclusion criterion, which may introduce survival bias and a shift in the captured symptomatic spectrum; the absence of key confounders (SpO2, LDH, formal severity scores) due to documentation limitations during the pandemic surge, with CRP, D-dimer, and ferritin missing in 37–66% of patients; no correction for multiple testing across the three correlated indices, such that broader ROC analyses should be regarded as exploratory; absence of internal validation, meaning that the Youden-derived optimal cut-off values represent derivation-only estimates and require external validation before clinical deployment; natural age-related CBC reference interval changes that may independently contribute to attenuated index performance in elderly patients [19]; and the non-monotonic SII–mortality relationship, suggesting that non-linear modeling approaches such as restricted cubic splines should be explored in future studies.

## 5. Conclusions

CBC-derived inflammatory indices such as SII, SIRI, and PIV demonstrate age-dependent prognostic performance for 30-day mortality in emergency department patients with COVID-19. Their predictive power is significantly modulated by age, performing well in younger adults (AUC 0.73 for SII, independent aOR 3.45) but poorly in the elderly (AUC 0.57, non-significant aOR 1.34), likely due to the effects of inflammaging, comorbidity burden, and competing risks.

Multivariate analysis confirmed that SII maintains independent prognostic value after adjusting for demographics and comorbidities (aOR 2.12), though this effect is primarily driven by its strong performance in younger patients. The high negative predictive values (>95%) support the use of these indices as rule-out tools, particularly for identifying low-risk younger patients suitable for outpatient management. However, the low positive predictive values indicate they should not be used in isolation for risk stratification.

Age-stratified triage algorithms that incorporate inflammatory indices for younger patients while prioritizing clinical comorbidities (particularly cardiovascular disease) for elderly patients may optimize ED resource allocation during pandemics. The persistent independent association of cardiovascular comorbidities with mortality highlights the need for integrated cardio-infectious disease protocols in pandemic response.

These findings provide preliminary hypothesis-generating insights into age-specific triage approaches that may be relevant not only to COVID-19 but also to future viral infections associated with systemic inflammatory responses. External validation in independent cohorts, incorporation of serial measurements, and development of non-linear models that account for age-inflammation interactions are warranted before these findings can be applied clinically.
